# CCL3L1 Copy Number Variation and Susceptibility to HIV-1 Infection: A Meta-Analysis

**DOI:** 10.1371/journal.pone.0015778

**Published:** 2010-12-30

**Authors:** SiJie Liu, Lei Yao, DongLin Ding, HuanZhang Zhu

**Affiliations:** The State Key Laboratory of Genetic Engineering, Institute of Genetics, School of Life Sciences, Fudan University, Shanghai, China; New York University, United States of America

## Abstract

**Background:**

Although several studies have investigated whether CCL3L1 copy number variation (CNV) influences the risk of HIV-1 infection, there are still no clear conclusions. Therefore, we performed a meta-analysis using two models to generate a more robust estimate of the association between CCL3L1 CNV and susceptibility to HIV-1 infection.

**Methods:**

We divided the cases and controls into two parts as individuals with CCL3L1 gene copy number (GCN) above the population specific median copy number (PMN) and individuals with CCL3L1 GCN below PMN, respectively. Odds ratios (ORs) with 95% confidence intervals (95% CIs) were given for the main analysis. We also conducted stratified analyses by ethnicity, age group and sample size. Relevant literatures were searched through PubMed and ISI Web of Knowledge up to March 2010.

**Results:**

In total, 9 studies with 2434 cases and 4029 controls were included. ORs for the main analysis were 1.35 (95% CI, 1.02–1.78, model: GCN ≤ PMN Vs. GCN > PMN) and 1.70 (95% CI, 1.30–2.23, model: GCN < PMN Vs. GCN ≥ PMN), respectively. Either in stratified analysis, statistically significant results can be detected in some subgroups.

**Conclusions:**

Our analyses indicate that CCL3L1 CNV is associated with susceptibility to HIV-1 infection. A lower copy number is associated with an increased risk of HIV-1 infection, while a higher copy number is associated with reduced risk for acquiring HIV-1.

## Introduction

Before 2009 acquired immunodeficiency syndrome (AIDS) has took over 25 million people's life, which have exceeded the death toll of the First World War. What is worse, nearly 33 million people suffered from HIV-1 infection [1]. It has been generally accepted that genetic variants among individuals can regulate HIV-1 cell entry, immune response and other factors that influence the susceptibility to HIV-1 infection, disease progressing and curative effects [2], [3]. Recently, CCL3L1 (human CC chemokine ligand 3-like 1) is recognized as one of the important genetic factors for HIV-1 infection.

CCL3L1, also known as macrophage-inflammatory protein 1α(MIP-1α), locates on chromosome 17q11.2 with clusters of cytokine genes. It can bind to several chemokine receptors including CCR5, which is one of the most important co-receptors for HIV-1 [4]. Binding of CCL3L1 to CCR5 may psychically block HIV-1 entry into cells by inhabiting the requisite co-receptor and suppress HIV viral replication in vitro [5]. Thus genetic variations among individuals that regulate the expression of CCL3L1 may influence susceptibility to HIV-1 infection.

CCL3L1 is highly variable in its copy number owing to having a hot spot for segmental duplications in the human genome [6]. Most individuals have 1 to 6 copies per diploid genome, while few individuals have 0 or more than 6 copies. Africans have larger copy number of CCL3L1 than non-Africans [4].

Recently, several studies reported that CCL3L1 CNV is tightly linked to HIV-1 susceptibility and processing a lower copy number of CCL3L1 in the geographic ancestral population is associated with increased risk of HIV-1 infection [7]–[12], whereas other studies didn't suggest this result [13]–[16]. Hence, we performed this meta-analysis of eligible studies to explore a more robust estimate of the association between CCL3L1 CNV and susceptibility to HIV-1 infection.

## Materials and Methods

### Literature research

Literatures were screened through PubMed and ISI Web of Knowledge up to March 2010, using the key words HIV-1, susceptibility, CCL3L1, MIP-1α in various combinations. Titles and/or abstracts were screened to estimate relevance of investigations. Full texts of primary selected literatures were downloaded for further study. Their references were hand-searched for potential related investigations. Articles were restricted to English language.

### Inclusion and exclusion criteria

The included investigations must accord with the following criteria: (1) Case-control studies reporting the association of CCL3L1 CNV and HIV-1 susceptibility; (2) Containing data of distribution of CCL3L1 GCN among the cohorts; (3) Published with English language. Major exclusion criteria as: (1) No sufficient data of CCL3L1 GCN distribution among the cohorts; (2) Reviews; (3) Duplication of previous studies.

### Data extraction and statistical analysis

One author (Liu) searched for eligible investigations according to the inclusion and exclusion criteria listed above. Study characteristics including authors, countries, year of publication, ethnicity, sample size of cohorts, distribution of CCL3L1 GCN among the cohorts were extracted by 2 authors (Liu and Yao) independently.

Crude ORs with 95% CIs were calculated to access the association of CCL3L1 CNV and HIV-1 susceptibility. Two genetic models were applied to analyse the distribution of CCL3L1 GCN among the cohorts. In the GCN ≤ PMN Vs. GCN > PMN model, cases and controls were divided into two parts as subjects with CCL3L1 GCN ≤ PMN and subjects with CCL3L1 GCN> PMN, respectively. And in the GCN < PMN Vs. GCN ≥ PMN model, cases and controls were divided into two parts as subjects with CCL3L1 GCN < PMN and subjects with CCL3L1 GCN ≥ PMN, respectively. Stratified analyses were performed by ethnicity, age group and sample size. Heterogeneity across studies was assessed with chi-square-based Q-test [17] and was considered significant if *p*<0.10, then the data were combined basing on the random-effects (Dersimonian and Laird) model [18]; otherwise the fixed-effects (Mantel and Haenszel) model was applied [19]. Publication bias was evaluated by Egger's and Begg's tests with visual inspection of funnel plots and was considered significant if *p*<0.05 [20], [21]. One-way sensitivity analyses were performed to examine the influence of individual studies on meta-analysis's results [22]. STATA version 10.0 (Stata Corporation, College station, TX) was used for all analyses.

## Results

**Figure 1:**
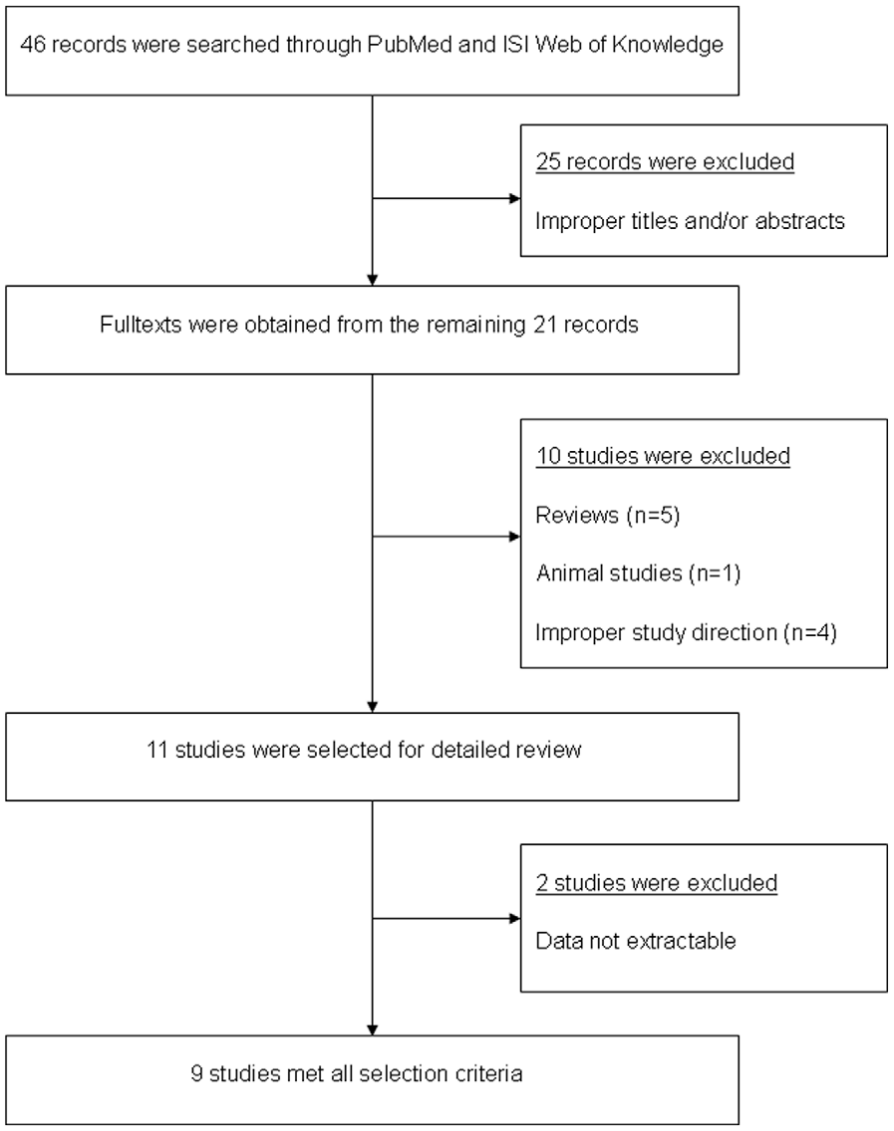
Selection process for study inclusion in the meta-analysis of CCL3L1 CNV and HIV-1 susceptibility.

Figure 1 indicates the selection process of literatures. We have 46 records searched through PubMed and ISI Web of Knowledge. After screening over titles and/or abstracts, full texts were obtained from the remaining 21 articles for further analysis [6]–[17], [23]–[32]. Among them 5 reviews were excluded [6], [24], [28], [30], [32], 1 study was excluded for its study object wasn't human being [26], 4 studies were excluded due to improper study direction (not aimed at the association of CCL3L1 CNV and HIV-1 susceptibility) [25], [27], [29], [31]. During the extraction of data, 2 studies were excluded due to insufficient data of CCL3L1 GCN distribution among the cohorts [13], [17].

Nine studies, involving 2434 HIV-1 infected patients and 4029 HIV-1 uninfected healthy donors, were included in this meta-analysis. Study sample size ranged from 120 to 3173 subjects. Study characteristics of the 9 eligible studies are summarized in Table S1. There're 5 studies involving subjects of African descendents [7], [8], [10], [14], [15], 5 studies involving subjects of Caucasian descendents [9], [10], [12], [14], [16]. Four studies reported the effect of CCL3L1 CNV on HIV-1 susceptibility in mother-to-child transmission [7]–[10], 6 studies were about adults [10]–[12], [14]–[16].

**Figure 2:**
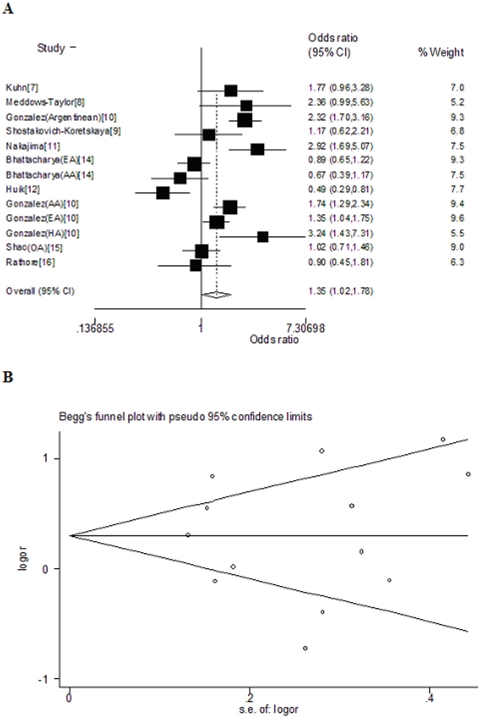
By using the GCN≤PMN Vs. GCN>PMN model, (a) forest plot of overall analysis; (b) funnel plot to detect publication bias in overall analysis. Data of studies involving mixed race was set apart according to different ethnicity. AA, African American; EA, European American; HA, Hispanic American; OA, Other American except African American.

In GCN ≤ PMN Vs. GCN > PMN model, the pooled OR was 1.35 (95% CI, 1.02–1.78, *P* = 0.000) (Figure 2a). Evaluation of publication bias by funnel plot (Figure 2b), Begg's test (*P* = 0.855) or Egger's test (*P* = 0.984) didn't reveal significant results.

**Figure 3:**
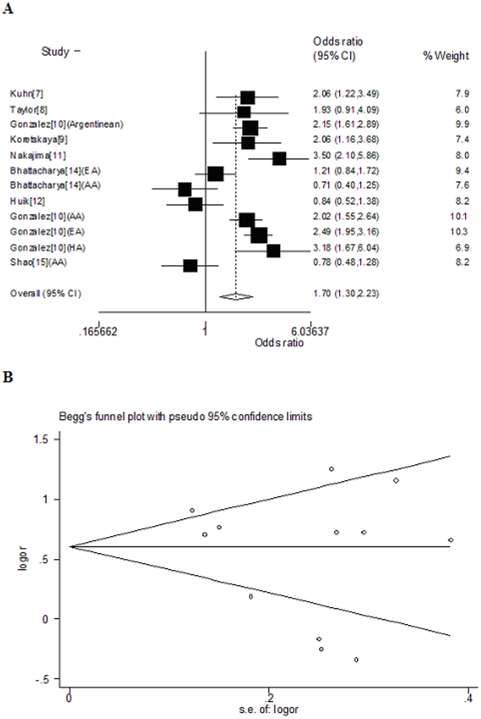
By using the GCN<PMN Vs. CCL3L1 GCN≥PMN model, (a) forest plot of overall analysis; (b) funnel plot to detect publication bias in overall analysis. Data of studies involving mixed race was set apart according to different ethnicity.

In GCN < PMN Vs. GCN ≥ PMN model, the pooled OR was 1.70 (95% CI, 1.30–2.23, *P* = 0.000) (Figure 3a). No asymmetry is observed in the funnel plot (Figure 3b), and neither Begg's test (*P* = 0.373) nor Egger's test (*P* = 0.324) suggested publication bias.

Also, we performed stratified analyses by ethnicity, sample size and age group to explore potential sources of heterogeneity and detailed relationship between CCL3L1 CNV and susceptibility to HIV-1 infection. The results were summarized in Table 1. Most of the them were consistent with the main analyses which indicated that CCL3L1 CNV have distinct impact on susceptibility to HIV-1 infection and a lower copy number of CCL3L1 in specific population is associated with higher risk of HIV-1 infection. Besides, results from subgroups stratified by age suggests hat CCL3L1 may play different roles on the resistance to HIV-1 infection between infants and adults [10]. Since there was only one study performed in Japanese, Indians and Argentineans respectively, further studies on Asians and South Americans were needed. The funnel plots of stratified analyses didn't show asymmetry (figure not shown), but the results of Egger's test suggested that publication bias was evident in sub-infants studies of the GCN<PMN Vs. GCN≥PMN model (*P* = 0.027).

**Table 1:** Results of meta-analysis for CCL3L1 CNV and HIV-1 susceptibility.

Study groups	GCN ≤ PMN Vs.GCN > PMN			GCN < PMN Vs.GCN ≥ PMN		
	N^a^	OR (95% CI)	*P*	N^a^	OR (95% CI)	*P*
Ethnicity						
African	4	1.44 (0.87–2.40)	0.016	5	1.35 (0.83–2.20)	0.000
Caucasian	6	1.08 (0.76–1.53)	0.001	5	1.73 (1.09–2.75)	0.000
Japanese	1	2.92 (1.69–5.07)	−	1	3.50 (2.10–5.86)	−
Sample size						
<500 subjects	8	1.35 (0.81–2.25)	0.000	8	1.58 (1.00–2.50)	0.000
>500 subjects	5	1.38 (1.00–1.92)	0.000	4	1.94 (1.48–2.55)	0.011
Age group						
Infants	4	2.01 (1.58–2.56)	0.274	4	2.10 (1.68–2.63)	0.994
Adults	9	1.19 (0.86–1.65)	0.000	8	1.55 (1.05–2.28)	0.000

a Number of comparisons.

Sensitive analysis was conducted by deleting one study at a time to examine the influence of individual data-set to the pooled ORs. All of the corresponding pooled ORs were not materially altered (Figure S1, S2).

## Discussion

Meta-analysis offers a powerful method to synthesize information of independent studies with similar target [33]. Considering a number of studies investigating CCL3L1 CNV and HIV-1 susceptibility have generated conflicting results, we performed this meta-analysis involving 9 eligible studies with 2434 cases and 4029 controls. The results suggested that CCL3L1 CNV is tightly linked to HIV-1 susceptibility. Individuals with lower copy number of CCL3L1 in the geographic ancestral population would be more susceptible to HIV-1 infection. The CCL3L-CCL4L region shows extensive architectural complexity, with smaller CNVs embedded within larger ones and with interindividual variation in breakpoints. Accounting for this genomic complexity is crucial for full interpretation of association studies, because the combinatorial content of CCL3L and CCL4L GCNs influences HIV/AIDS susceptibility. Methodological and epidemiological confounders and failure to account for the genomic complexity of the CCL3L-CCL4L locus underlie the lack of associations reported by several studies [34].

It is generally accepted that variations of gene copy number is a genetic determinant of phenotypic variation [6]. Since CCL3L1 encodes the natural ligand of CCR5 and increasing copy number of CCL3L1 down-regulates the expression of CCR5 on the cell surface [10], the HIV-1 entry-dependent effect of CCL3L1 on CCR5 used to be accepted as the major mechanism that inhibiting HIV-1 infection. Recent studies suggested that genotypes of CCL3L1-CCR5 influence cell-mediated immunity of individuals [35]. Individuals with higher copy number of CCL3L1 have greater CD4^+^ and CD8^+^  T cell response to HIV-1 gag protein [6], [29]. These findings indicated that both viral entry dependent effect and independent effect might explain the result of this meta-analysis.

Although the mechanism of CCL3L1 inhibiting HIV-1 infection has not been fully explored, outcomes of this meta-analysis confirmed that CCL3L1 CNV have distinct impact on HIV-1 susceptibility and a lower copy number of CCL3L1 in specific population is associated with higher risk of HIV-1 infection. Several factors might underlie the lack of observed association between CCL3L1 CNV and HIV-1 susceptibility. First, relatively small sample size would lead to results with too limited statistical power to detect possible relationship between genetic polymorphism and disease risk in genetic association studies [36]. Generally, large sample size is required for genetic association studies of single gene and disease susceptibility depending on the prevalence of the implicated polymorphism, since susceptibility of specific disease is always determined by numbers of genes [37]. Especially for people with relatively lower copy number of CCL3L1 like Caucasians, insufficient sample size would have generated a fluctuated risk estimate [38]. Second, selection of cases might have significant influence on the results. Infection with HIV-1 can exert a negative selective pressure on individuals with lower copy number of CCL3L1. Over time, the prevalence of cases with CCL3L1 copy number equal or greater than specific population median will increase because subjects with lower copy number of CCL3L1 progressed to AIDS or death more rapidly [10]. Thus, if selected cases weren't at an early stage of infection, similar distribution of CCL3L1 copy number would probably be observed in HIV-1 infected and uninfected subjects. Fourth, the susceptibility to HIV-1 infection is affected by a combination of genes besides CCL3L1. Nevertheless, few studies had dissected the combinatorial genomic complexity posed by varying proportions of distinct CCL3L and CCL4L genes among individuals [40]. Finally, Although most of the studies adopted similar real-time PCR method (Taqman assay) to measure the copy number of CCL3L1 in samples, minor methodological discrepancies of different studies as differences in DNA concentration and differences in primer and probe dye chemistry might have affected the results [39].

Some limitations of this meta-analysis should be acknowledged. First, publication bias was evident in subgroup of infants/hospital-based cases and hospital-based controls (*P* = 0.027) and subgroup of population-based cases and population-based controls (*P* = 0.014). The potential reason may be studies with advantage results would be accepted and published more easily. Thus, more studies are required for further analysis. Second, HIV-1 of R4 strain take advantage of CXCR4 as co-receptor but not CCR5. Therefore, virus entry-dependent effect of CCL3L1 won't affect HIV-1 infection of R4 strain. Nevertheless the newly discovered virus entry-independent effect might not be influenced despite HIV-1 virus type since most of the patients were infected by R5 strain. It is unlikely that virus of X4 strain would significantly affect the results. Third, in stratified analysis by ethnicity, the included studies regarded mostly Europeans and Africans. Only 300 subjects of Japanese and 511 subjects of Indians were included in published studies originated from Asia, which were too small to have enough statistical power. Additional studies are warranted to evaluate the association of CCL3L1 CNV and HIV-1 susceptibility in different ethnicities, especially in Asians. Forth, GCN estimates by currently quantitative PCR assays suffer from relatively low precision and accuracy with increasing gene copy number. In studies of Caucasians or other ethnicities that the median copy number of CCL3L1 is 2 or less, the methodological shortcomings should be a minor concern. But it might be more troublesome in studies of Africans with higher copy number [38]. Newer method with higher precision and accuracy will be helpful for a more exact estimate of the association between CCL3L1 CNV and HIV-1 susceptibility. Finally, controls of most studies were derived from healthy individuals. For studies concerning disease susceptibility, it'll be more proper to take samples from HIV-1-exposed seronegative (HES) individuals as controls. Considered the difficulty of obtaining samples from HES individuals, further studies of larger scale is required.

In conclusion, our meta-analysis involving 9 eligible studies with a total of more than 6000 subjects indicated significant association between CCL3L1 CNV and HIV-1 susceptibility. A lower copy number of CCL3L1 in the geographic ancestral population is associated with higher risk of HIV-1 infection. For further studies, it seems to be crucial to define precisely the genomic structure, taking into account the specific combination of the distinct genes within a CNV region [40], and considering (1) mechanism of CCL3L1 against HIV-1 infection; (2) discrepancies of methods on measuring CCL3L1 gene copy number; (3) CCL3L1's effect on Asians; (4) HES individuals as controls.

## Supporting Information

Figure S1 **Sensitive test of the GCN≤PMN Vs. GCN>PMN model.**
(TIF)

Figure S2 **Sensitive test of the GCN<PMN Vs. CCL3L1 GCN≥PMN model.**
(TIF)

Table S1 **Characteristics of studies included in the meta-analysis.**
(DOC)
