# Supplementary figures and images for: CCL3L1 Copy Number Variation and Susceptibility to HIV-1 Infection: A Meta-Analysis
Source: PLoS One. 2010 Dec 30;5(12):e15778. doi: 10.1371/journal.pone.0015778 (PMC3012711; doi:10.1371/journal.pone.0015778)

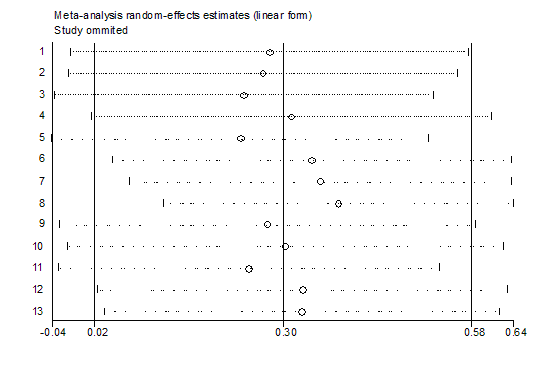

Supplement: Figure S1 — Sensitive test of the GCN≤PMN Vs. GCN>PMN model. (TIF) [file pone.0015778.s001.tif]

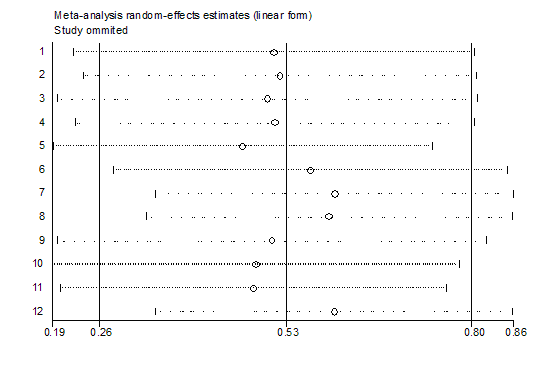

Supplement: Figure S2 — Sensitive test of the GCN<PMN Vs. CCL3L1 GCN≥PMN model. (TIF) [file pone.0015778.s002.tif]
