# Supplementary material for: CCL3L1 Copy Number Variation and Susceptibility to HIV-1 Infection: A Meta-Analysis
Source: PLoS One. 2010 Dec 30;5(12):e15778. doi: 10.1371/journal.pone.0015778 (PMC3012711; doi:10.1371/journal.pone.0015778)
Supplement: Table S1 — Characteristics of studies included in the meta-analysis. (DOC) [file pone.0015778.s003.doc]

| Study (year) | Country | Ethnicity | CCL3L1 median copy number  (case/control) | Sample size  (case/control) | M_L_/M_H_ ^a^ | N­_L_/N_H_ ^b^ | X_L_/X_H_ ^c^ | Y_L_/Y_H_ ^d^ |
| --- | --- | --- | --- | --- | --- | --- | --- | --- |
| Kuhn[7] (2007) | South Africa | African | 4/5 | 79/235 | 63/16 | 162/73 | 50/29 | 107/128 |
| Taylor[8] (2006) | South Africa | African | 4/5 | 46/74 | 37/9 | 47/27 | 28/18 | 33/41 |
| Shostakovich-Koretskaya [9] (2009) | Ukraine | Ukrainian | 2/2 | 178/120 | 156/26 | 100/20 | 52/126 | 20/100 |
| Gonzalez[10] (2005) | America | Argentinean | 2/2 | 407/395 | 319/88 | 241/154 | 186/221 | 111/284 |
|  |  | African-American | 3/4 | 409/497 | 318/91 | 332/165 | 240/169 | 205/292 |
|  |  | European-American | 2/2 | 620/675 | 491/129 | 498/177 | 260/360 | 152/523 |
|  |  | Hispanic-American | 2/3 | 69/101 | 60/9 | 68/33 | 46/23 | 39/62 |
| Nikajima[11] (2007) | Japan | Japanese | 3/4 | 95/205 | 73/22 | 73/22 | 64/31 | 76/129 |
| Huik[12] (2010) | Estonia | Caucasian | 2/2 | 166/208 | 122/44 | 177/31 | 35/131 | 50/158 |
| Bhattacharya[14] (2009) | America | African-American | 3/4 | 402/338 | 277/125 | 241/97 | 91/311 | 66/272 |
|  |  | European-American | 2/2 | 142/82 | 71/71 | 49/33 | 46/96 | 33/49 |
| Shao[15] (2007) | America | African-American | 4/4 | 161/177 | - | - | 54/107 | 46/71 |
|  |  | Other American | 3/3 | 66/65 | 38/28 | 39/26 | - | - |
| Rathore[16] (2009) | India | Indian | 2/2 | 196/315 | 103/93 | 164/151 | - | - |

**Table S1. Characteristics of studies included in the meta-analysis.**

^a^ Number of cases with CCL3L1 GCN ≤ PMN / Number of cases with CCL3L1 GCN ＞ PMN.

^b^ Number of controls with CCL3L1 GCN ≤ PMN / Number of controls with CCL3L1 GCN ＞ PMN.

^c^ Number of cases with CCL3L1 GCN ＜ PMN / Number of cases with CCL3L1 GCN ≥ PMN.

^d^ Number of controls with CCL3L1 GCN ＜ PMN / Number of controls with CCL3L1 GCN ≥ PMN.
